# Open source drug discovery – A limited tutorial

**DOI:** 10.1017/S0031182013001121

**Published:** 2013-08-28

**Authors:** MURRAY N. ROBERTSON, PAUL M. YLIOJA, ALICE E. WILLIAMSON, MICHAEL WOELFLE, MICHAEL ROBINS, KATRINA A. BADIOLA, PAUL WILLIS, PIERO OLLIARO, TIMOTHY N. C. WELLS, MATTHEW H. TODD

**Affiliations:** 1 School of Chemistry, The University of Sydney, Sydney, NSW 2006, Australia; 2 Medicines for Malaria Venture, PO Box 1826, 20 rte de Pré-Bois, 1215 Geneva 15, Switzerland; 3 UNICEF/UNDP/World Bank/WHO Special Programme for Research and Training in Tropical Diseases (TDR), World Health Organization, Avenue Appia 20, 1211 Geneva 27, Switzerland

**Keywords:** Drug discovery, open source, open science, schistosomiasis, malaria, collaboration

## Abstract

Open science is a new concept for the practice of experimental laboratory-based research, such as drug discovery. The authors have recently gained experience in how to run such projects and here describe some straightforward steps others may wish to take towards more openness in their own research programmes. Existing and inexpensive online tools can solve many challenges, while some psychological barriers to the free sharing of all data and ideas are more substantial.

## INTRODUCTION

Open Science is a way of practising science in which all data and ideas are freely shared, anyone may participate and there are no patents (Gowers and Nielsen, 2009; Nielsen, 2011; Woelfle *et al.* 2011*a*, *b*; Reaves *et al.* 2012). It presents a number of advantages over more traditional methods of carrying out research, such as speed, involvement of a wide network of experts with no prior contact with the core team, robustness of solutions arising from continual peer review, release of both positive and negative data for reuse by others and a reduction in unnecessary duplication of research worldwide.

The methodology of open science mimics the open source movement in software development that has achieved significant commercial success with products such as industry-leading web browsers (e.g. Firefox) and operating systems (e.g. Linux). Open science is, however, a new concept, and there is some degree of variation in adherence to these core principles (Årdal and Røttingen, 2012), and some confusion with other, completely distinct, ideas such as open innovation (Hunter and Stephens, 2010). Open science has now been applied by us to drug synthesis and drug discovery in the area of tropical diseases (Woelfle *et al.* 2011*a*, *b*). This article describes lessons learned in the course of two specific open projects, intended as a guide for those wishing to employ similar methods in their own research. Both projects carried with them the challenge of being based in experimental science, i.e. where physical objects had to be made, handled and shared. For a non-experimental example of open science in mathematics, see Gowers and Nielsen, 2009; for excellent examples of crowdsourcing online see Land *et al.* (2008) and Cooper *et al.* (2010).

The first project involved the discovery of a way to generate the chiral drug praziquantel (PZQ) as a single enantiomer (Woelfle *et al.* 2011*a*, *b*). The widespread use of PZQ is hampered by side effects and a bitter taste caused by the distomer (Meyer *et al.* 2009). A strategic goal of the Special Programme for Research and Training in Tropical Diseases (TDR) of the World Health Organisation had been to perform a ‘chiral switch’ on PZQ that would result in a pill containing the (*R*)-enantiomer while keeping the price low. An open approach to this project was started in 2006, but a funded kernel of effort began in the laboratory in January 2010. The inputs of the broader community led to both changes in direction of the research and an acceleration of the science, leading to the publication in 2011 of a solution to the problem suitable for scale-up.

While this was an important proof-of-concept, it begged the question of whether similar ideas could be applied to drug *discovery*, where the intellectual property landscape is more complicated, and the long-term funding regime less clear; such an idea had been mooted or started but a full campaign never tried (Maurer *et al.* 2004; Bradley *et al.* 2008). There have been major moves to make the drug discovery process more collaborative, such as *via* Product Development Partnerships like the Medicines for Malaria Venture (MMV) and the Drugs for Neglected Diseases Initiative (DNDi) or other ventures (Norman *et al.* 2011). The sharing of chemical compounds to stimulate research in malaria and other neglected diseases (the ‘Malaria Box’) has even been pioneered by (MMV Spangenberg *et al.* 2013).

An open source drug discovery consortium in malaria was begun and is now in the second year of its existence (http://opensourcemalaria.org). It is currently centred in Sydney (Australia) but has involvement from various laboratories around the world, including links to a group with similar ideals in India that has to date focused more on tuberculosis chemo- and bioinformatics (Bhardwaj *et al.* 2011; http://malaria.osdd.net/). The aim of the project is to take hits (molecules with some cellular activity) such as those placed in the public domain from the GlaxoSmithKline Tres Cantos phenotypic screen (Gamo *et al.* 2010) and prosecute a drug discovery project with the aim of generating a compound entering Phase I clinical trials.

These two projects are centred on basic experimental scientific research, in that the same day-to-day questions are being asked, and the same theoretical approaches (e.g. general medicinal chemistry methods) are followed as for regular research projects. It is rather the shift to working entirely in the public domain that is so radical and, it turns out, productive. This article is an ‘under-the-hood’ description of how such projects function, intended for those considering a move towards open source science, but who are unsure how such projects can work. Before turning to the details it is important first to be clear on the philosophy that underpins open science. This philosophy was formulated during the PZQ project, and true to form has been in the public domain since the start of the malaria project, stated as Six Laws (http://www.thesynapticleap.org/node/343):

First Law: All data are open and all ideas are shared;
Second Law: Anyone can take part at any level of the project;
Third Law: There will be no patents;
Fourth Law: Suggestions are the best form of criticism;
Fifth Law: Public discussion is much more valuable than private email;
Sixth Law: The project is bigger than, and is not owned by, any given laboratory.

Should any of these principles be unacceptable, it would be advisable to steer clear of open science in practice. While these laws may be an initial psychological/organizational/legal challenge, it has been found that there are simple practical steps that anyone may take to work in this way that require zero capital investment.

## DISCUSSION

### Methodology of open research

#### Electronic laboratory notebook

**Fig. 1.**
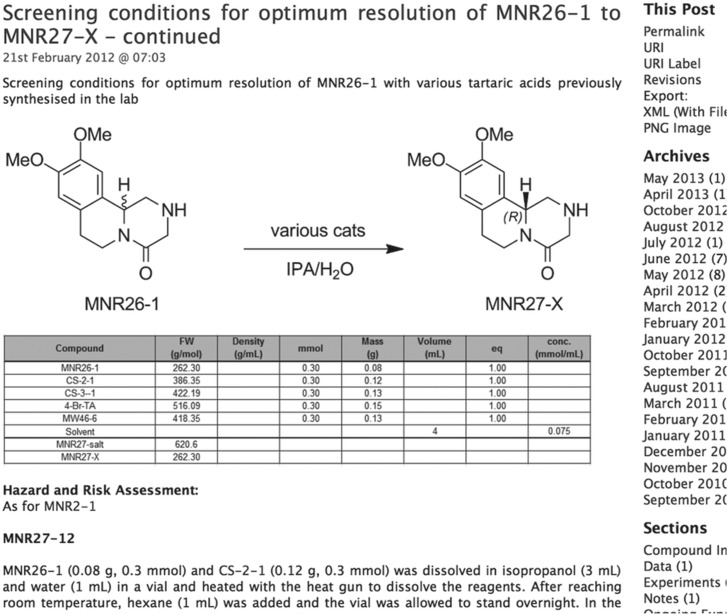
A sample page from an electronic lab notebook created with the open source software, Labtrove.

The bedrock of an open project is a means to share all primary data. This requires that the laboratory notebook moves from paper to digital and from the benchtop to the internet. While there are many commercial electronic laboratory notebooks (ELNs) we have to date adopted an open source ELN because we did not want to rely on the commercial health of an organization for the survival of the data, and because it was important that potential future collaborators (particularly from developing nations) would not need to purchase software in order to participate. The solution we chose (Labtrove, Fig. 1; http://www.labtrove.org/) may be downloaded and installed locally; as an open source product Labtrove may be modified and the modified code may be made freely available to the community, as one of us has already done (Modtrove: https://github.com/miike/modtrove).

Each researcher can maintain a separate notebook within a project, or laboratory notebooks can be shared. The ELN should be kept like a regular laboratory notebook, including for the posting of hazard assessments and authorizations. It is simple to maintain links between related experiments and links to relevant primary literature. Raw data can be posted alongside human-readable processed versions of data, meaning that all data are maintained in one place and backed up. The ELN provides permanent links to individual experiments that may be used in papers to refer to research on an experiment-by-experiment basis (Woelfle *et al.* 2011*b*). All changes to the ELN are recorded and automatically time-stamped (a current and significant weakness of paper laboratory notebooks). To reduce the barrier to participation, login (required if one wishes to comment on another's work) is simple *via* a number of common authentication mechanisms but is not necessary to read the content.

We have found the ability to post images has been beneficial in terms of encouraging reproducibility. Images are also inserted to illustrate chemical reactions or structures. Tables of data may be inserted, but they currently lack spreadsheet functionality that would be useful for trivial calculations. Required improvements to the software like this can be submitted to the community as a feature request for future releases.

The ELN itself may be searched, but to ensure the maximum discoverability of the notebook itself by people outside the project it is important that the content is understood by search engines. In the case of molecules, machine-readable text-strings (using the SMILEs or InChI systems, that are easy to generate) (Southan, 2013) can be inserted manually in ELN entries.

A more detailed description of this online electronic laboratory notebook will shortly be published elsewhere. It should be emphasised that the deposition of all primary data is the one central element to any open science endeavour, without which a truly collaborative effort will not function. Not least, the availability of raw data permits objective quality control of all contributions.

#### Data management

**Fig. 2.**
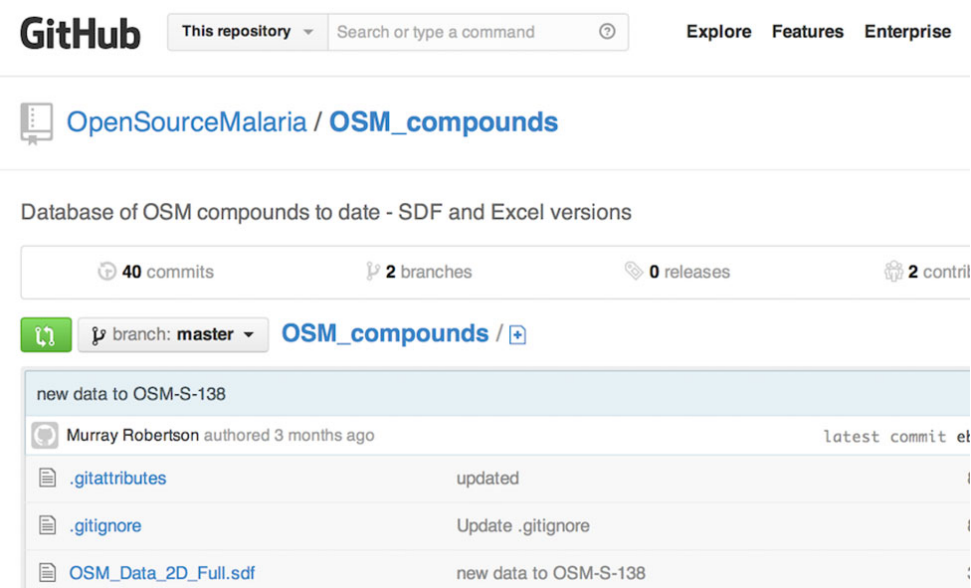
Management of updatable data files using existing software designed for managing coding projects.

The primary data generated in the open source drug discovery for malaria project consist of structures with physical and biological properties. In a closed project, one might keep such data in a spreadsheet or electronic database, but when the data are to be shared, and remain modifiable by the community, this is not so straightforward. We found that a widely-used online spreadsheet application (Google Docs) was adequate for smaller sheets of data, but found the system strained when it was required to retrieve images of multiple molecular structures in the course of loading. Ultimately, we adopted an approach borrowed from the open source software movement where sophisticated websites exist for management of coding projects and which are suitable for management of communal data, in this case an SD file that contains all chemical information and may be downloaded, modified and re-uploaded (Fig. 2). (Ram, 2013; https://github.com/OpenSourceMalaria/OSM_compounds) The file is discoverable by search engines.

**Fig. 3.**
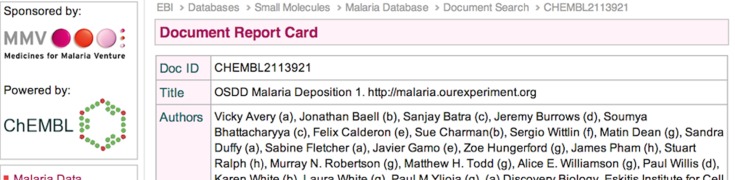
Deposition of data in openly-available online databases enhances discoverability.

Local data management for the project is necessary, but in the longer term the data need to be maximally discoverable and searchable, meaning they should be curated to a level that is probably best left to specialists and, in the case of the malaria project, the data have been batch-uploaded to the chemical informatics database of the European Molecular Biology Laboratory, ChEMBL (Fig. 3) (https://www.ebi.ac.uk/chembl/malaria/doc/inspect/CHEMBL2113921; Avery *et al*. 2013). Besides the presentation of data in the most useable format, the dataset itself gains permanence, for example *via* a Digital Object Identifier (DOI). In future a more automated approach to feeding data from local to larger databases will be needed. There are other tools we have not employed that others might find valuable for the sharing of chemical and biological data (e.g. Figshare, Dryad, Pubchem, ChemSpider and other non-profit/commercial databases) or places where the data might usefully be incorporated (e.g. OpenPhacts). There are also valuable tools to prompt the machine discovery of chemical information in web pages that can then feed into the aforementioned resources (e.g. Chemicalize).

#### Beyond primary data – coordination tools

Scientific research happens in a variety of ways beyond the laboratory notebook, and it is important for open projects to capture not just data and workflow but strategy. The primary means of doing this is to post project updates on a more familiar blogging environment that allows input by others with a low barrier to entry. The Synaptic Leap website (http://www.thesynapticleap.org/) fulfils this role as a coordination space and is built on the popular open source standard known as Drupal, but any blogging platform would suffice here as a first approximation. A website like this also functions as a marketing tool, to act as a central place people can remember to go for the latest updates. More widely, a function of the central blog can be to allow anyone to run their own open science projects, as is the case in The Synaptic Leap.

**Fig. 4.**
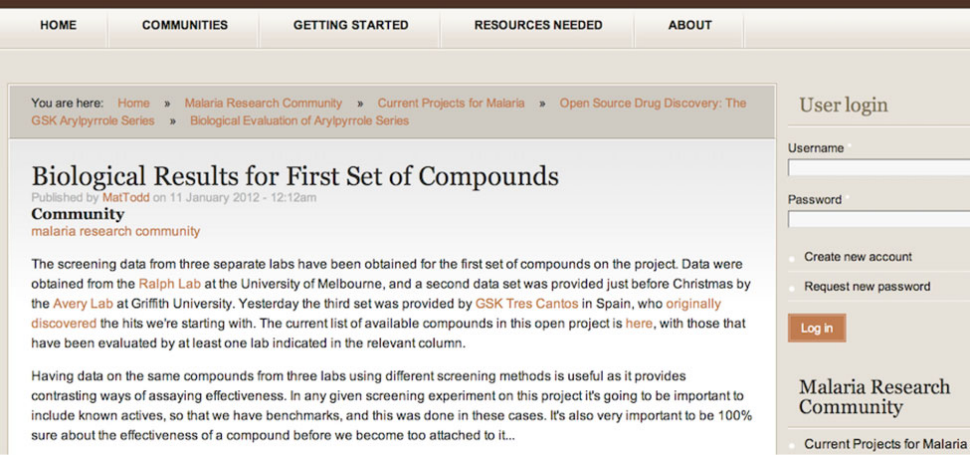
The Synaptic Leap, a coordination site allowing project discussion and updates.

Project updates act as a means to distil results in smaller units than a full write up in a paper, while also bringing people new to the project up to speed. Updates are written by the researcher who did the work, not necessarily the person in charge of the project. Users of the coordination site can post *via* an account name or anonymously. Conducting science in this way has a democratising or levelling effect that undercuts the traditional academic monikers – thus for example in comments under a post in the malaria project (Fig. 4) (http://www.thesynapticleap.org/node/367) an undergraduate student and a professor who had never met were able to discuss openly a point of theory about the data without it ever mattering who was who.

**Fig. 5.**
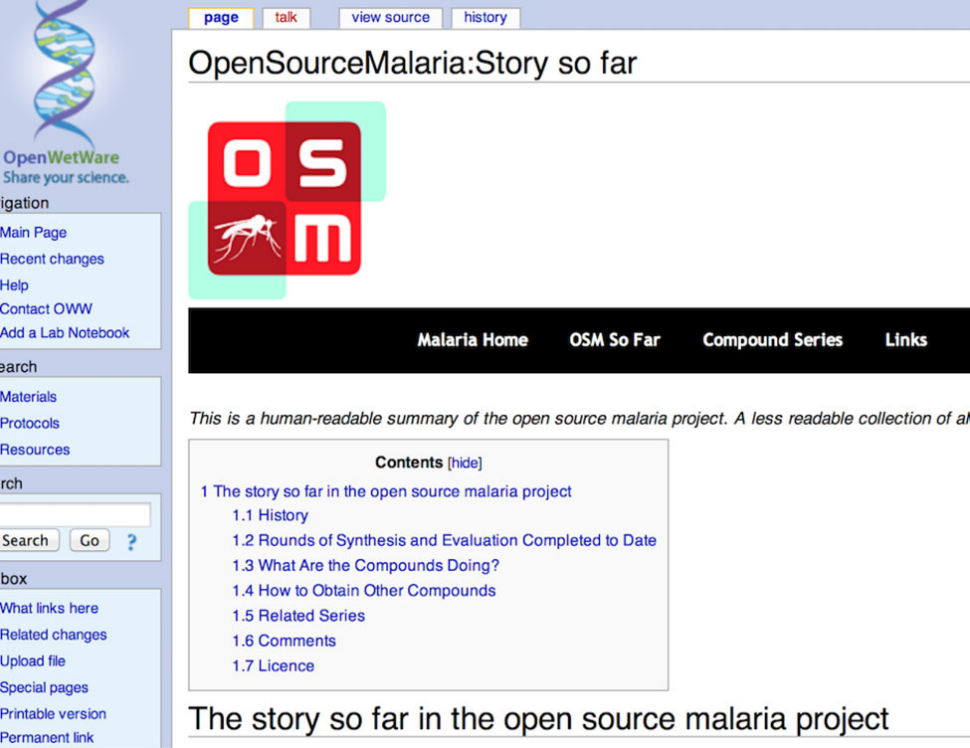
A project wiki can act as a project status page.

Active coordination like this is important, but many blog posts become out of date and are intended mainly for temporary consultations. It is useful to maintain one continually updated page that describes project status – a page that does not grow linearly, but is changed only in response to achievements. A wiki can be useful for this and there are many suitable providers, such as OpenWetWare, used in both our current projects. Ease of use is slightly lower than for a blog, and there is much less visible discussion on a wiki, but for the core team of a project this is a simple way to update key discoveries and to provide one page that newcomers can be directed to that describes the history of the project. The malaria project's ‘Story-so-Far’ page (Fig. 5) (http://openwetware.org/wiki/OpenSourceMalaria:Story_so_far) has to date been visited around six thousand times. A wiki can also be a useful place to draft papers collaboratively, owing to most wiki sites maintaining revision histories, records of provenance of contributions and a ‘talk’ page where geographically separated contributors can address what needs to be written. There will, however, come a point where the paper needs to be adapted to the usual requirements for submission to a journal for peer review, and typically this will require lengthy formatting changes, e.g. in the way references are handled, that slightly reduces the usefulness of wikis in this role. Academic theses, should they arise from the project, can be shared in the public domain *via* a number of means such as University repositories or other services, and may gain significant exposure as a result (Cronshaw, 2012).

#### Tools to enhance community input

An open project requires momentum to be successful and this momentum is provided by participants who can arise from the core team or from the wider community. Those in the wider community can become involved only if they know of the project's existence. It is thus important to publicise what is happening on the project, and this means posting updates, questions or requests for help in places where people gather. It also means *detailed public discussion of the project before publication*, one of the most serious psychological barriers to many scientists in traditional disciplines, yet one of the most obvious steps to those with a background in software development.

**Fig. 6.**
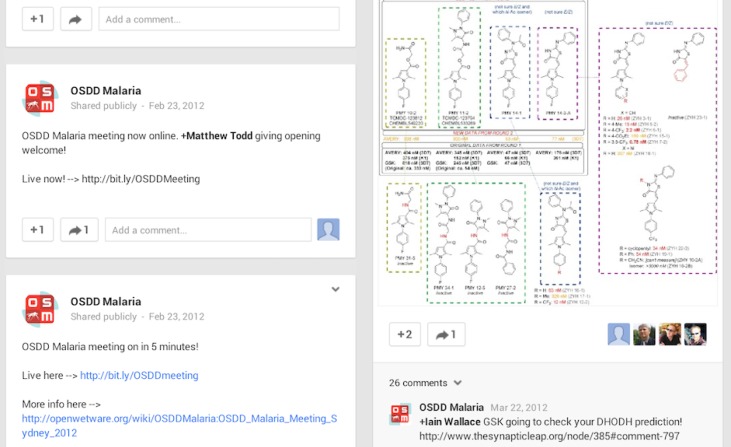
Example Google+ post in the open source drug discovery for malaria project.

For our projects, we have found that various freely-available tools provide new and useful functionality for the coordination of experimental science. One of the most promising recent additions has been Google+ (Fig. 6), which hosts pages or groups for project-specific discussions, allows the simple posting of pictures or event invitations to meetings, and is a platform with a large pre-existing public user base. Other popular social networking platforms could also fulfil this role. It was found in the PZQ project that a more focused professional networking site (LinkedIn) was a productive way to reach a specialised target audience, since there are groups there with specific focus on areas such as process chemistry or medicinal chemistry; it helps if these groups are open, as opposed to members-only. These sites can be useful for short-term pushes to solve small problems; the discussion lasts only for a short time since activity becomes buried quickly by newer items and only those people who originally participated in a discussion will be alerted to future posts. If a specific issue is identified, and if others can engage through providing ‘microcontributions’ then significant successes can be achieved in the short term. It should be stressed that these platforms are being run by commercial organizations with no guarantee of long-term survival; one lives with the risk of platform collapse in the future, while gaining the benefit of a large user base that would otherwise be difficult to generate.

For the community to be able to help in guiding the research through timely inputs, it is important they are aware of where the project is headed in the long (e.g. month-quarter) term. Their participation in online project meetings is desirable, and may be achieved to some extent with freely available teleconferencing tools (e.g. Skype, Google Hangout) but this is one area where we have resorted to a commercial solution which minimises latency during the meeting, permits multiple participants from diverse time-zones and allows the recording of the meeting (Adobe Connect). The recording should be used for the creation of meeting minutes that are posted online alongside the recording (e.g. on popular sites such as YouTube). The use of blogs has proven to be successful in generating valuable suggestions and discussions here. It is important that an open project is seen to be responsive to this varied input. It is also important that key strategies for the research period following any meetings are clearly decided and described; this opportunity to agree future strategy collectively has been a particular benefit of online project meetings in terms of maintaining focus and momentum.

Smaller project alerts can be managed with popular platforms (such as Twitter) (https://twitter.com/O_S_M), for example in the posting of a link to a new project update, or the asking of a short question. Again, the power of such platforms for generating help with a certain project-related issue lies in their popularity, rather than in their perceived academic rigour. As with any such site we have found it is important for the updates to be human-derived (interactive) rather than made in broadcast mode by a feed or a machine – i.e. should you ask a question of the community, it is both good manners and more productive if there is a human being ready to interact with the responses.

#### Miscellaneous

Other tools exist that might become useful to open projects, but which we have not used extensively to date. Collaborative management of references is possible (e.g. with Mendeley), collaborative discussion of scientific problems is possible on a number of platforms (e.g. ResearchGate) and there is work underway to aggregate topic-specific scientific content from the internet (Ekins *et al.* 2012). There are sites known to be effective in community problem solving in software development (e.g. Stackoverflow) that have created sophisticated metrics for measurement of contributions that have not yet impacted significantly on the workflow of open science projects. The manipulation of data between formats will become challenging once a dataset becomes large, and will eventually begin to require specialist tools, for which there are robust open source solutions (e.g. Knime); much of this activity will be served by valuable emerging open standards for data (O'Boyle *et al.* 2011).

The management of physical samples is an interesting challenge for experimental open science. In some senses this is a straightforward problem: regular mail and courier services are adequate for movement of samples. But in a broader sense, the global management of physical objects in a productive manner is a huge unsolved problem. If one thinks of the number of samples of molecules contained in fridges and freezers of industrial and academic laboratories worldwide, one realises there is a great untapped resource that could have a significant impact on open science projects with the provision of effective information management. For example, Synthetic Laboratory A may possess a collection of unpublished molecules structurally similar to a hit molecule of great interest to Medicinal Chemistry Laboratory B. These molecules may all be ineffective but at least Laboratory B would not need to spend time on laborious resynthesis if it knew of Laboratory A's compound archive. All that is required to transform this situation, besides the shift to a more open mindset, is suitable coordination of these samples to match needs with supply, and a great deal of scientific progress could be made with greater efficiency – what one of us termed the ‘Molecular Craigslist’ problem (http://intermolecular.wordpress.com/2012/04/20/molecular-craigslist/).

#### Origin of contributions

With all these tools in place, and once an open project is generating data, is it reasonable to expect people to contribute? Based on our recent experience, and the truly enormous efforts in open source software development worldwide, the answer is yes. One should not expect to need thousands of participants for a project – a handful of motivated, knowledgeable participants, particularly those able to contribute experimental resources, can make a big difference. Where might one expect to find such help and contributions? This is not easy to predict. With a regular flow of experimental data, regular project updates, a clear identification of the needs of a project that is helpfully split into small tasks, help might appear from anywhere. With a clear incentivization (authorship, community acclaim or good public relations) and a low barrier to entry, it is simple for potential contributors to act in their own interests and contribute. Inputs from industry, for example, might be expected for selfish reasons of good public relations or demonstration of core technical competence to potential future clients. As the move towards greater openness starts to take hold in science, the pool of interested contributors should grow, as should the ease of finding the most relevant people or, more importantly, the ease with which suitable experts can find open projects on which they can have an impact.

#### What's missing?

After several years of assessment of how to run experimental open science projects, it is clear to the authors that while there are many useful tools that permit science to be carried out productively in this way, still there are many important components lacking. Some of these relate to logistics, i.e. how to manage distributed projects. The tools available allow a project to get off the ground, but they are not yet ideal. For example, the ELN we have employed lacks several features, ranging from the simple (effective notifications of comments on entries) to the complex (the impossibility of drawing and editing a chemical structure on a webpage). It is important to keep the action items of what is currently needed clearly visible on a project ‘Landing Page’ where fresh content is placed automatically – a current need for the malaria project (Note added in proof: this page now exists [http://opensourcemalaria.org]). The creation of new entries on our Synaptic Leap blogging platform has been an issue, due to the eccentric abilities of the site to handle images and formatting; these can usually be overcome, but if one presents a barrier to project entry like this one can expect mildly enthusiastic people to drift away or to clog up the project mechanism by requesting core team members to troubleshoot others' entries. One of the key advantages of using open source software for such projects is that the software can be adapted by the community as the needs of the open science projects become clearer and more sophisticated in the future.

The stability of the scientific record increasingly depends on the longevity of web-based resources (Schultheiss *et al.* 2011). While a diversity of websites can fulfil different project roles, as the project grows so does the number of ‘dead’ links that have not been adequately updated to reflect project progress. Those wishing to run open projects must commit time and resources to the management of websites.

Specifically with regards to chemical synthesis and biological evaluation, there is an issue of users outside the project finding the relevant science. As mentioned above, chemical content can be manually added to web pages through text strings, or through manual prompting of online tools, but it is clearly going to be important for chemical content to be retrieved, understood and managed across the web by machines in the generation of freely-available databases. Though this remains a daunting problem, the benefit would be enormous, since this would create an internet that could inform the chemical reader who is working on related chemistry or compounds at the present moment, rather than once an article appears in the academic literature many months later.

An issue remains that is relevant to the psychology of participants too. There are concerns about working in the public domain, ranging from the embarrassment of being wrong, through to a concern that preliminary data could be taken as ‘final’ or ‘confirmed’ data. There is thus a typical desire to interact, particularly by newcomers, by email, which generates a closed line of communication that often then needs to be converted to one in the public domain. It is the authors' opinion that such concerns and practices will diminish, but these will certainly require careful management by anyone considering starting a scientific project in the public gaze. Researchers will also obviously need to clarify, and possibly challenge, the default practices of their employers regarding any intellectual property implications of the work being pursued.

### Residual questions

#### Will I get scooped?

Possibly. If that can happen in a short timeframe, it might be time to select another research problem. However, one should realise that open deposition of results on a webpage (backed up by primary research data) is the quickest dissemination mechanism possible. Though not without risk, this may favour the junior academic who is willing to experiment with new dissemination methods. Authors attempting to scoop your work then need to acknowledge your prior art. Those authors could claim ignorance of your work; this is difficult to prove or disprove, but with the power of current search engines, and their ubiquitous use, such a claim is weakening.

Participants in an open project should hope that their work is widely read, and widely used by others. The licence adopted should therefore be clearly stated somewhere on each online resource. Readers are directed to valuable resources describing the licences that could be considered for open endeavours (http://pantonprinciples.org/; Williams *et al.* 2012; Wilbanks 2013); a Creative Commons CC-BY licence (http://creativecommons.org/licenses/by/3.0/) was adopted in both projects described in this article and serves the purpose well – in essence the licence states that others may use any part of the project in their own work, even to make money, provided the open projects are cited. This promotes reuse of the data and ideas of a project (including to stimulate commercial activity) while maintaining proper academic standards of attribution.

#### If I release everything, surely that prevents commercialization of my work?

Possibly (Caulfield *et al*. 2012). The lack of a clear answer on this issue was one of the major drivers in setting up the open source malaria consortium in the first place. Open source software underpins technologies of enormous commercial significance, many of which are trademarked or copyrighted, rather than patented. To the specific assertion of whether a patent is needed to create a drug, space does not permit a proper answer to this interesting question, but powerful arguments against the need for patents have already been published by others (Boldrin and Levine, 2008). The arguments may alter depending on a drug development candidate's Net Present Value, i.e. the net costs of developing *vs* selling the drug (DiMasi *et al*. 2003; Svennebring and Wikberg, 2013). However, clearly much of the basic science that underpins drug discovery is amenable to an open approach (with a prominent example being the open access chemical probes developed by the Structural Genomics Consortium) (Edwards *et al*. 2009).

#### How do I publish open science?

Many journals are now open access or provide open access options, and these obviously need to be used in order to prevent a total philosophical own-goal. However, many such journals will also accept research that has already appeared in the public domain – for example the PZQ project (Woelfle *et al*. 2011*b*) was published in a high-impact open access journal despite all the data having been deposited in openly available ELNs. A peer-reviewed research paper plays a crucial role as a summary of an open project, upon the reaching of a certain milestone. The journal article is important since it is cited, permanent and a declaration by the open project that part of the research is complete, and has been peer-reviewed. Publishing in the traditional way remains an important component of reputation building for junior scientists and career development for senior scientists. The mechanism of publishing in open access journals may depend on the funding agency supporting the work, or the country of residence of the corresponding author, and any associated costs will need to be factored into the project plan at the outset. An intriguing possibility to promote the practice of open science might be for publishers to discount the fees associated with such projects.

#### How do I fund an open science project?

If this all sounds like an attractive and productive way to work, the final issue is how to get it funded. The tools above can be adopted with virtually zero capital investment, and many research organizations should be willing to provide the modest IT support necessary to run the infrastructure (e.g. a server for the ELN, and some guarantee of data backup). Salaries for those driving the core of a project would help with that project's momentum, but open projects flourish because of the contributions of others, and these can arise from people in a wide variety of circumstances, ranging from research professionals contributing because the project aligns with their core intellectual or commercial interests, through to students contributing in their spare time.

It is the authors' belief that research is moving towards a more open era, and that it will become increasingly difficult to justify research in basic science that does not attempt to make an effort to involve extra research expertise from around the world, to publish negative data, to ensure maximum data reuse and to avoid unnecessary duplication of effort. Funding agencies should see the added value of funding a project kernel to which others, not funded by the core grant, can contribute. Beyond mandating open access to research papers, such agencies should be aware of the extra downstream benefits of open data stimulating further research outputs (Royal Society, 2012). This is perhaps best exemplified by the Human Genome Project, where the costs of the project are dwarfed by the downstream economic impact of the open data produced (Battelle, 2011). Beyond open data lies open source, where participants may help guide and improve the research itself before it is carried out – the subject of this article.

The kernel funding for the two open projects described above arose from a combination of industry/NGO funds matched with government funding. An open source project is a highly distributed and mobile research project, in which people give time and resources when it suits them, but the composition of the team constantly changes. There are therefore few ‘start-up’ costs to get the project team moving since resources that currently exist will be employed, and should unplanned needs arise, new contributors can be brought in for small contributions without the requirement for long-term commitments. This creates a ‘nimble’ team that can adapt quickly to changes, and which can be broader than the circle of acquaintances of the chief investigators. Again, though this can represent something of a challenge to the traditional management structure of a research project, the benefits have, in our experience, more than compensated and can be proposed in grant applications.

Readers are welcome to contact the authors to discuss any aspects of running open science projects along the lines described above. Avoidance of bilateral channels such as email would be much appreciated. (The corresponding author for example can be reached on Google+ through a search of his name, or on Twitter at @mattoddchem).
